# γ-H2AX Kinetics as a Novel Approach to High Content Screening for Small Molecule Radiosensitizers

**DOI:** 10.1371/journal.pone.0038465

**Published:** 2012-06-29

**Authors:** Shibo Fu, Ying Yang, Das Tirtha, Yun Yen, Bing-sen Zhou, Ming-Ming Zhou, Michael Ohlmeyer, Eric C. Ko, Ross Cagan, Barry S. Rosenstein, Shu-hsia Chen, Johnny Kao

**Affiliations:** 1 Department of Radiation Oncology, Mount Sinai School of Medicine, New York, New York, United States of America; 2 Department of Radiation Biology, Jilin University School of Public Health, Changchun, China; 3 Department of Dermatology, Columbia University, New York, New York, United States of America; 4 Department of Developmental and Regenerative Biology, Mount Sinai School of Medicine, New York, New York, United States of America; 5 Department of Medical Oncology and Therapeutic Research, City of Hope National Medical Center, Duarte, California, United States of America; 6 Department of Structural and Chemical Biology, Mount Sinai School of Medicine, New York, New York, United States of America; 7 Department of Oncological Sciences, Mount Sinai School of Medicine, New York, New York, United States of America; 8 Department of Radiation Oncology, Good Samaritan Hospital Medical Center, West Islip, New York, United States of America; Dresden University of Technology, Germany

## Abstract

**Background:**

Persistence of γ-H2AX after ionizing radiation (IR) or drug therapy is a robust reporter of unrepaired DNA double strand breaks in treated cells.

**Methods:**

DU-145 prostate cancer cells were treated with a chemical library ±IR and assayed for persistence of γ-H2AX using an automated 96-well immunocytochemistry assay at 4 hours after treatment. Hits that resulted in persistence of γ-H2AX foci were tested for effects on cell survival. The molecular targets of hits were validated by molecular, genetic and biochemical assays and *in vivo* activity was tested in a validated *Drosophila* cancer model.

**Results:**

We identified 2 compounds, MS0019266 and MS0017509, which markedly increased persistence of γ-H2AX, apoptosis and radiosensitization in DU-145 cells. Chemical evaluation demonstrated that both compounds exhibited structurally similar and biochemical assays confirmed that these compounds inhibit ribonucleotide reductase. DNA microarray analysis and immunoblotting demonstrates that MS0019266 significantly decreased polo-like kinase 1 gene and protein expression. MS0019266 demonstrated *in vivo* antitumor activity without significant whole organism toxicity.

**Conclusions:**

MS0019266 and MS0017509 are promising compounds that may be candidates for further development as radiosensitizing compounds as inhibitors of ribonucleotide reductase.

## Introduction

Despite the central role of DNA damage repair in determining efficacy of radiation therapy (RT) or cytotoxic chemotherapy, developing specific inhibitors of DNA damage repair is a relatively unexplored area of research [1]. In the pharmaceutical and biotechnology industries, high throughput screening is a central function in the drug discovery process [2]. To date, this strategy has only recently been applied to experimental radiotherapy [3], [4]. The most common high-throughput screens are biochemical assays that screen for compounds that interact with an isolated protein on an assay plate [2]. In contrast, a cell-based approach provides insight into the permeability profile of active compounds and enables the identification of compounds with unique mechanisms of action [5]. Charged-coupled device (CCD) camera- based plate imaging systems allow for high throughput quantitation of cellular and subcellular fluorescence in whole cells [6]. These high content cell based assays allow for screening compounds that impact cellular functions, such as cell cycle, cell motility, apoptosis and DNA repair [6]. Current disadvantages of this approach include limited throughput related to incompatibility of some steps of a complex screening procedure with full automation, the relatively high cost of reagents and data-management issues. Despite these technical hurdles, there is significant interest in applying high content screening to primary drug screening [5].

Exposure to IR and many chemotherapy agents, including DNA synthesis inhibitors, DNA alkylators and topoisomerase I inhibitors, result in DNA double strand breaks (DSB) [7], [8], [9]. A single unrepaired DSB may result in cell death demonstrating the critically important role of DNA damage repair in maintaining genomic integrity [10]. An emerging concept is that the physiological target of IR is not DNA alone but rather DNA in the three-dimensional context of chromatin within a complex and highly regulated protein-DNA structure [11]. ATM and related kinases phosphorylate Serine 139 on H2AX to form foci of γ-H2AX immunoreactivity at DNA DSB sites that can be visualized by light microscopy [12], [13]. The phosphorylation of H2AX at DSBs has been implicated in the timely recruitment and/or retention of DNA repair and checkpoint proteins such BRCA1, MRE11/RAD50/Nbs1 complex, MDC1 and 53 bp1 to sites of DNA damage [9], [14], [15], [16]. Downstream signal transduction pathways may result in DNA damage repair (homologous recombination, non-homologous end joining), cell cycle arrest or apoptosis [17]. γ-H2AX interacts with NuA4, INO80 and SWRC, proteins that play a key role in chromatin remodeling and histone acetylation. The cohesion complex, which joins sister chromatin allowing for efficient homologous recombination repair of DSB appear to localize to sites of DSB via interaction with the INO80 complex [18], [19]. Persistence of γ-H2AX between 3 to 24 hours following experimental treatment is strongly associated with unrepaired DNA DSB and sensitivity to DNA damaging therapies [20]. Based on these data, we performed a proof-of-concept screen to identify agents that increased persistence of γ-H2AX at 4 hours after drug treatment and ionizing radiation by inhibiting DNA damage repair. Using a reverse chemical genetics approach, a secondary focus of this study is to elucidate the molecular target of lead compounds identified by the screen [6].

## Results

### High-content Screening for Compounds that Effectively Induce γ-H2AX and Reduce Tumor Cell Viability

**Figure 1:**
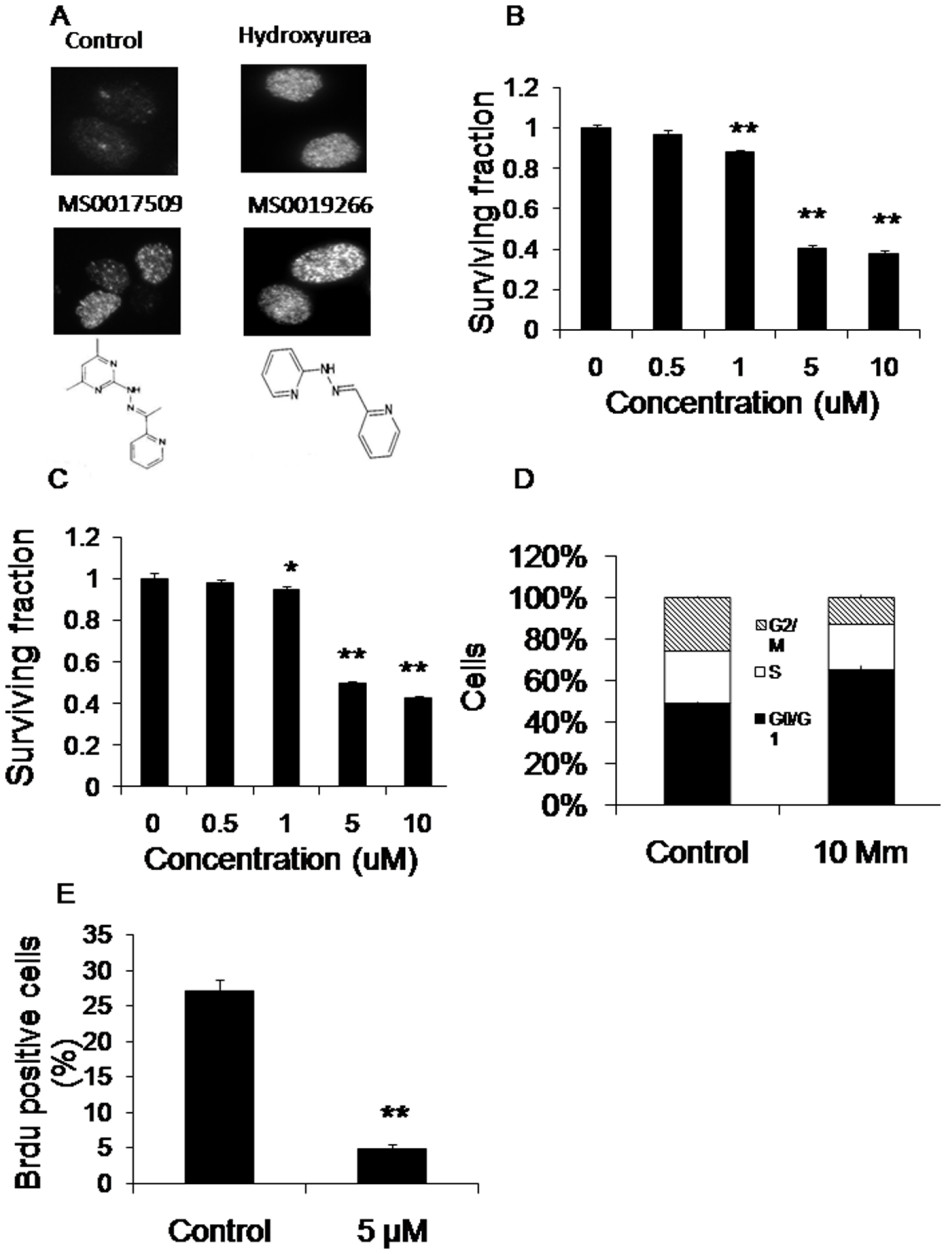
Identification of MS0019266 and MS 0017509 as inhibitors of DNA damage repair. A) DU-145 prostate cancer cells were treated with mixtures of 30 compounds from the 14,400 compound chemical library (10 µM) for 4 hours and stained for γ-H2AX and then imaged with a fluorescence microscope. MS0019266 (10 µM) and MS0017509 (10 µM) were identified as individual compounds that significantly increased γ-H2AX within 4 hours of treatment. Hydroxyurea (200 µM) for 4 hours was used as a positive control. B and C) Both MS0019266 (10 µM) and MS0017509 (10 µM) treated DU-145 cells were assessed for cell viability using MTS assay. After 72 hours of treatment with MS0019266 and MS0017509 respectively resulted in a significant decrease of cell viability in a dose-dependent compared to vehicle controls. Results are reported as means ±SEM and normalized to control (*P<0.05, **P<0.01; vs. control as determined by two-tailed Student’s *t*-test). D) Cell cycle analysis by flow cytometry demonstrated cells arrest in G1 phase at 24 hours after MS0019266 (5 µM) treatment. E) Cell proliferation was assessed by BrdU incorporation assay. After 24 hours of treatment, DU145 cells were incubated with BrdU solution at final concentration of 10 µM for 30 min. MS0019266 (5 µM) markedly reduced BrdU incorporation consistent with a marked decrease in DNA synthesis.

We screened 14,400 compounds in 480 mixtures of 30 compounds for the specific phenotype of increased γ-H2AX in DU-145 prostate cancer cells at 4 hours after drug therapy alone or in combination with 2 Gy of radiation. We identified 2 compound mixtures that had an especially strong effect on inducing γ-H2AX foci at a dose of 10 µM for 4 hours with or without radiation (Figure 1a). An additional 12 compound mixtures demonstrated a weaker positive effect. Only the 2 compound mixtures that significantly induced γ-H2AX reduce cell viability, while the 12 compound mixtures that weakly induced γ-H2AX did not significantly affect cell viability.

To identify the specific active compounds from these two mixtures, we then tested mixtures of 10 compounds derived from the parent 30 compound mixtures for induction γ-H2AX 4 hours after treatment. Based on these results, we ultimately tested individual compounds for increased persistence of γ-H2AX and decreased tumor cell viability. Through this unbiased screening process, we identified the 2 most active small molecules as MS0019266 (2-pyridinecarbaldehyde 2-pyridinylhydrazone; molecular weight 198.2) and MS0017509 (1-(2-pyridinyl) ethanone (4,6-dimethyl-2-pyrimidinyl)hydrazone); molecular weight 241.3) (Figure 1A). Both MS0019266 and MS0017509 significantly inhibited DU-145 tumor cell viability at doses as low as 5 µM (Figures 1B and C). Further studies demonstrated that SCC-25, SCC-61 and SQ-20B head and neck cancer cells are sensitive to MS0019266 at doses as low as 0.2 µM while PC-3 prostate cancer cells as low as 5 µM (Figures S1 and S2).

### MS0019266 Markedly Reduces DNA Synthesis, Induces Apoptosis and Increases Persistence of γ-H2AX in Combination with Ionizing Radiation

**Figure 2:**
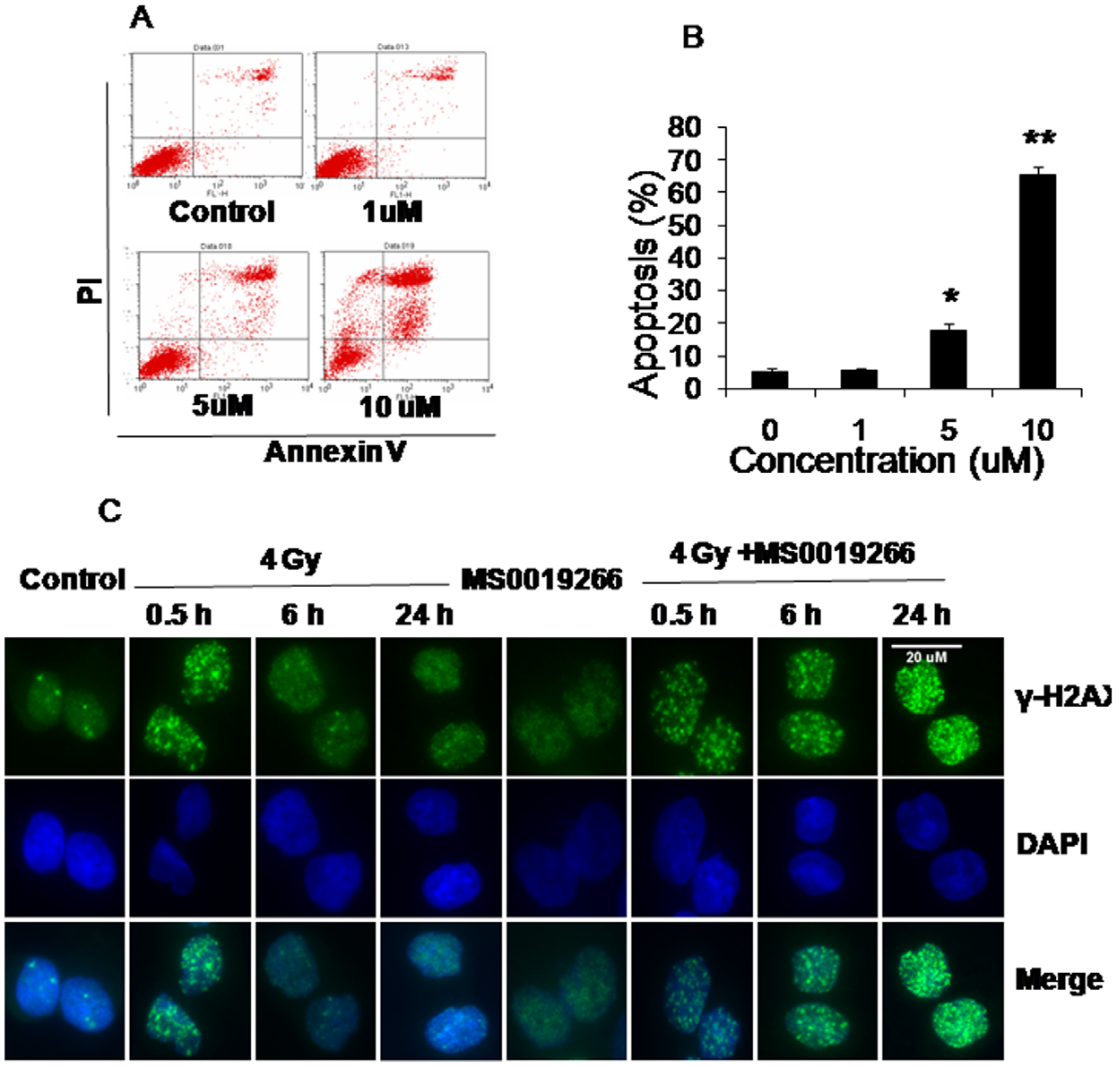
Effect of MS0019266 on apoptosis and DNA damage repair. A and B) Effect of MS0019266 on apoptosis. Annexin V level after 72 h of treatment with MS0019266 (ranging from 1–10 µM) in DU145 cells was examined by flow cytometry. MS0019266 significantly increased apoptosis in DU145 cells at doses ≥5 µM. C) Effect of MS0019266 on induction of γ-H2AX foci as measured by immunocytochemistry. Combined treatment with MS0019266 (10 µM) and 4 Gy ionizing radiation significantly increases nuclear γ-H2AX foci at 6 to 24 hours after treatment compared to radiation alone.

Evaluation of the chemical structures of MS0019266 and MS0017509 demonstrates shared structural motifs (Figure 1a). Since MS0019266 is slightly more active than MS0017509 according to MTS data, we first characterized the effects of MS0019266 on DU-145 cell biology. Based on cell cycle analysis, treatment with MS0019266 (5 µM) resulted in a slight G1/S arrest (Figure 1D). Assessment of BrdU incorporation confirmed a significant reduction in DNA synthesis during S-phase in MS0019266 treated cells (Figure 1E). Flow cytometric analysis demonstrates significant induction of apoptosis in DU-145 cells at doses ≥5 µM (Figure 2A and B). In combination with 4 Gy ionizing radiation, MS0019266 markedly increases the induction and persistence of γ-H2AX foci at 6 to 24 hours after treatment (Figure 2C).

### In-vitro Radiosensitizing Effects of MS0019266 and MS0017509

**Figure 3:**
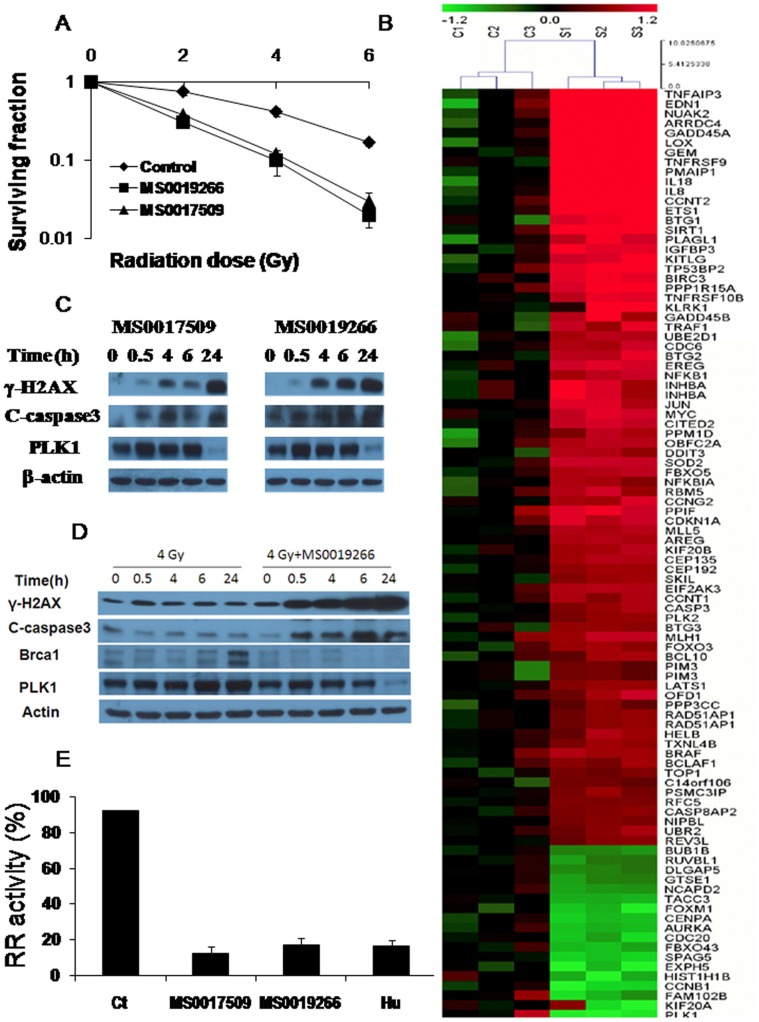
MS0019266 and MS0017509 enhance tumor cell radiosensitivity and specifically targets ribonucleotide reductase and polo-like kinase 1. A) Colony formation assay performed on DU145 cells. Pretreatment with MS0019266 and MS0017509 (1 µM for 72 hours) significantly reduced clonogenic survival in DU-145 prostate cancer cells subsequently treated with ionizing radiation administered 24 hours after drug administration. Following 72 hours of drug treatment, cells were switched to drug free media. Results are the average of experiment and each experiment was done in triplicate (n = 3). * denotes p<0.05 and ** denotes p<0.01 vs. vehicle control. B) Gene expression changes following treatment with MS0019266 as assessed by gene expression microarray. Genes showing differential expression between MS0019266 (5 µM for 8 hours) treated cells and vehicle treated controls. Upregulated genes are shown in red and downregulated genes are shown in green. C) γ-H2AX cleaved, caspase3 and PLK1 expression were examined by immunoblotting. Administration of MS0019266 (10 µM) and MS0017509 (10 µM) for 4 hours resulted in significant accumulation of γ-H2AX and cleaved caspase3 over time in DU-145 cells. In contrast, PLK1 is markedly decreased at 24 hours after drug treatment. D) Combined treatment with MS0019266 (10 µM for 4 hours) and ionizing radiation resulted in more significant accumulation of γ-H2AX compared to either MS0019266 or radiation alone. PLK1 is also markedly decreased at 24 hours after combined treatment with radiation and MS0019266. Further, treatment of irradiated cells with MS0019266 (10 µM for 4 hours) markedly reduced the accumulation of radiation-induced BRCA1 repair protein at 6 and 24 hours following ionizing radiation. E) MS0017509 and MS0019266 are potent inhibitors of *in vitro* ribonucleotide reductase activity. Administration of MS0019266 (10 µM) and MS0017509 (10 µM) for 6 hours prior to CDP assay resulted in a similar reduction of ribonucleotide reductase activity as hydroxyurea (200 µM) in treated DU-145 cells.

To determine the effects of MS0019266 and MS0017509 on prostate cancer radiosensitivity, clonogenic assays were performed on the DU-145 cell line. As shown in Fig.3A, pretreatment DU145 cells with1 µM MS0019266 and MS0017509 respectively, a dose at which MS0019266 and MS0017509 did not cause any loss of viability, induced significant radiosensitization compared to vehicle control alone (p<0.05 at all dose levels). Corresponding dose enhancement ratios (DER) was 2.3 and 2.1 respectively. This effect was also confirmed in SQ20B head and neck cancer cells at a sub-micromolar dose of MS0019266 (Figure S3).

### Effect of MS0019266 on Genes Related to Cell Cycle, DNA Repair and Apoptosis

Since the molecular targets of MS0019266 are unknown, we performed comparative global gene expression analysis on DU-145 cells at 8 hours after treatment with MS0019266 (5 µM) (Figure 3B). MS0019266 resulted in the most significant reduction in polo-like kinase (PLK-1; 2.7 fold reduction) gene expression (Table 1). There was also evidence of a 1.8 to 2.4 fold reduction in the expression of genes related to cell cycle regulation and mitosis including kinesin family member 20a, cyclin B1 and aurora kinase A and a 4-fold increase in GADD45A expression. Genes significantly increased by MS0019266 appeared non-specifically related to treatment-related cell stress including tumor necrosis factor associated protein 3, endothelin 1, NUAK2 and lysyl oxidase (4 to 8.6 fold increase).

**Table 1:** Genes Differentially Expressed in MS0019266-treated vs. vehicle control treated DU-145 cells.

Gene description	Fold change	Gene symbol
*Upregulated (≥4 fold increase)*		
Tumor necrosis factor, alpha-induced protein 3	8.6	TNFAIP3
Endothelin 1	6.8	EDN1
NUAK family, SNF1-like kinase, 2	6.1	NUAK2
Arrestin domain containing 4	4.7	ARRDC4
Growth arrest and DNA-damage-inducible, alpha	4.0	GADD45A
Lysyl oxidase	4.0	LOX
GTP binding protein overexpressed in skeletal muscle	4.0	GEM
*Downregulated (≥2 fold decrease)*		
Polo-like kinase 1	2.7	PLK1
Kinesin family member 20A	2.4	KIF20A
Family with sequence similarity 102, member B	2.2	FAM102B
Cyclin B1	2.1	CCNB1
Histone cluster 1, H1b	2.1	HIST1H1B
Exophilin 5	2.0	EXPH5

### MS0019266 and MS0017509 Affects Protein Levels Related to DNA Damage Repair and Apoptosis

To validate phenotypic functional and gene expression data in treated cells, we performed immunoblotting to assess effects on protein levels. To assess the effects of MS0019266 and MS0017509 (10 µM for 4 hours) on DNA damage repair, we determined the kinetics of γ-H2AX induction and accumulation in DU-145 cells. Although evidence of γ-H2AX induction occurs as early as 30 minutes after treatment, significant accumulation of γ-H2AX occurs at 4 hours and continues through at least 24 hours after drug treatment (Figure 3C). Concurrent administration of ionizing radiation and MS0019266 markedly amplifies this effect strongly implicating inhibition of DNA double strand break repair is a potential mechanism of action (Figure 3D). Following 4 Gy ionizing radiation alone, BRCA1 accumulates 6 to 24 hours of ionizing radiation as γ-H2AX foci are being cleared (Figures 2C and 3D). The accumulation of BRCA1 at 6 and 24 hours following ionizing radiation is abrogated by treatment with MS0019266 (Figure 3D). Taken together, these data suggest a robust that MS0019266 interferes with DNA double strand break repair following ionizing radiation. To confirm the effect of MS0019266 or MS0017509 on apoptosis, we determined the kinetics of cleaved caspase-3 accumulation in DU-145 cell. Cleaved caspase-3 accumulates in treated cells starting 4 to 6 hours after treatment with MS0019266 or MS0017509 (Figure 3C and Figure 3D).

### MS0019266 and MS0017509 Reduces PLK-1 Protein Expression

Gene expression data suggests that PLK-1 is a potential target of MS0019266. Immunoblotting of MS0019266 and MS0017509 (10 µM for 4 hours) demonstrates a potent reduction in PLK-1 protein levels at 24 hours after treatment, with or without ionizing radiation (Figures 3C and D).

### MS0019266 and MS0017509 Inhibit Ribonucleotide Reductase Activity

Based on SciFinder search, chemical structures similar to MS0019266 and MS0017509, such as 3-AP (triapine) have been reported to inhibit ribonucleotide reductase (Figure S4) [21], [22]. MS0019266 has no detectable effects on ribonucleotide reductase M1 and ribonucleotide reductase M2 protein or gene expression (data not shown). However, DU-145 cells treated with MS0019266 (10 µM), MS0017509 (10 µM) and hydroxyurea (200 µM) for 6 hours were all highly effective at inhibiting *in vitro* ribonucelotide reductase activity compared to controls (Figure 3E). Both MS0019266 (10 µM) and MS0017509 (10 µM) were at least as effective as hydroxyurea (200 µM).

### MS0019266 Suppresses Tumorigenesis in a *Drosophila* Model of MEN2

**Figure 4:**
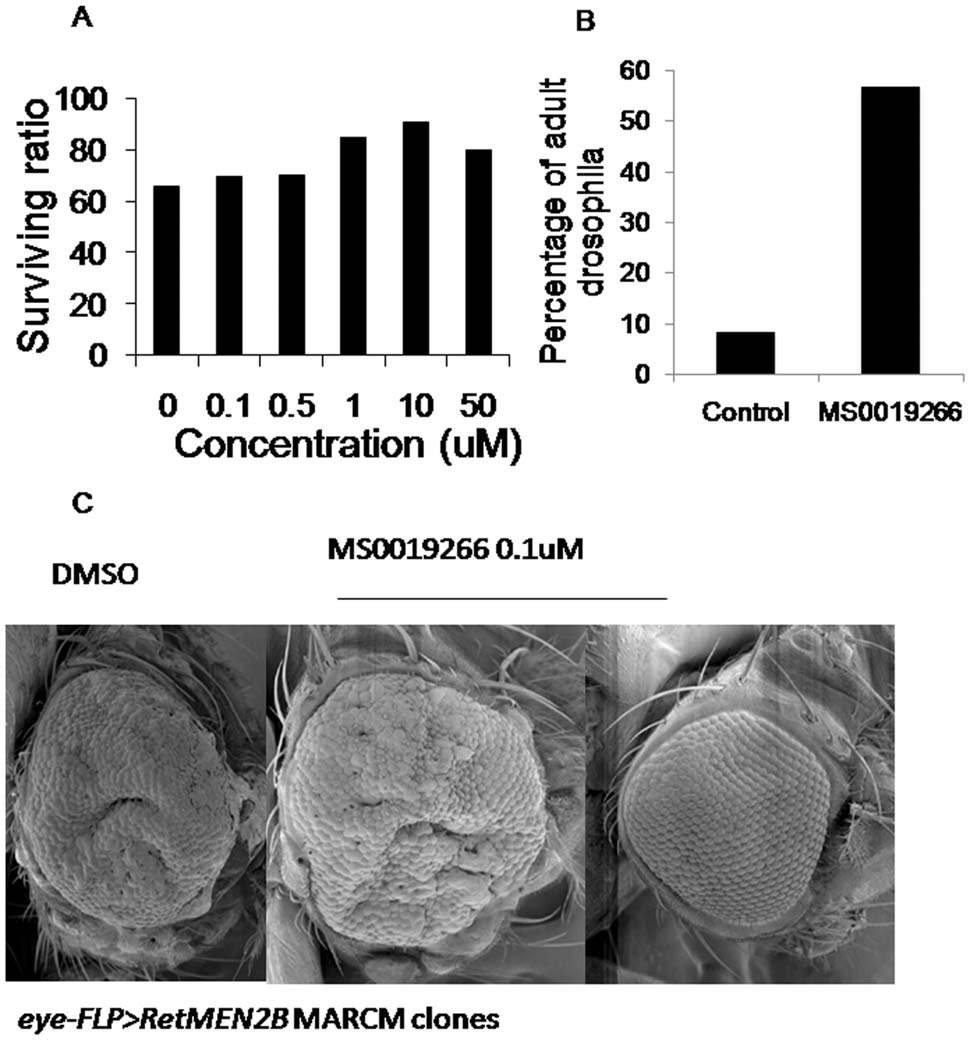
Results of MS0019266 administration in a *Drosophila* model of MEN2. A) MS0019266 treated fly embryos demonstrates a dose dependent increase in survival to pupae stage in *Drosophila* suggesting antitumor efficacy. B) MS0019266 (0.1 to 50 µM) markedly increased *Drosophila* surviving to adult stage suggesting that treated animals can tolerate doses into the micromolar range without lethal toxicity. C) MS0019266 suppresses development of eye tumors related to RetMEN2B *in vivo.* Scanning electron micrographs of adult *Drosophila* eyes from flies fed on control food (left panel) or food containing 100 nM MS0019266 (center and right panels). Large eye tumors developed in 100% of controls and 90% of MS0019266 treated flies. For 10% of flies fed MS0019266 during development, normal eye development was restored demonstrating effective abrogation of RET-mediated tumor development.

Using the GAL4-UAS system, oncogenic Ret isoforms can be expressed simultaneously in various tissues of the developing *Drosophila* larvae (*ptc> dRet^MEN2B^* ). This leads to only ∼55% of the *ptc> dRet^MEN2B^* embryos reaching pupal stages and none to adulthood due to lethal progression of ret-mediated eye tumors. Known ret inhibitors, like Sorafenib and ZD6474, can suppress the *dRet^MEN2B^*-induced rough eye phenotype and increase proportion of animals reaching pupal and adult stages. When larvae expressing oncogenic Ret (*ptc> dRet^MEN2B^* ) were fed compound MS0019266 (100 nM to 50 µM), there was a significant increase in viability of these organisms, and resulted in a significantly higher proportion reaching pupae (∼70% to ∼90%) and adult stages (∼25% to ∼90%) compared to vehicle control (Figure 4a). Development of pupae appeared to be dose dependent up to doses of 10 µM. In contrast, development of adult Drosophila was not dose dependent but was increased 7 fold compared to vehicle only controls (Figure 4b). This suggests that compound MS0019266 can suppress oncogenic signaling *in-vivo* at doses not associated with lethal toxicity. Treatment with 100 nM or 10 µM of MS0019266 was able to partially suppress the hyper-proliferative capacity of *dRet^MEN2B^* expressing cells, which were eliminated from the adult eye in 5 to 10% of treated flies (Figure 4c).

## Discussion

Approximately 1 in 4 deaths in the United States are caused by cancer that is refractory to therapy, suggesting that further improvements in anticancer therapy are urgently needed [23], [24]. One approach to overcoming radiation resistance is utilizing combined chemotherapy and radiation, which improves survival compared to RT alone in locally advanced brain, cervix, lung, head and neck, rectal, esophagus, bladder and anal cancers [25]. The rationale for using cytotoxic chemotherapy as a radiosensitizer is based on the rationale that additional DNA damage may lower the threshold of cell death by ionizing radiation (IR). Although clinically significant radiosensitization has been achieved with cytotoxic chemotherapy, this approach is limited by lack of tumor cell selectivity, resulting in increased toxicity [24].

To address the unmet need for developing new treatments to further improve cancer treatment, such as small molecule inhibitors, new and innovative approaches are needed. Despite the central role of DNA damage repair in determining response to radiation, therapies that inhibit this process have not been successfully developed as clinically useful radiosensitizers [1]. For instance, wortmannin, caffeine or LY294002 have not been clinically useful radiosensitizers due to lack of tumor cell specificity resulting in an unacceptable toxicity profiles [26]. Recently PARP inhibitors have been shown to be highly effective in tumors with an underlying susceptibility to DNA damage, such as BRCA1 and BRCA2 mutation related cancers [27]. Further progress in this area was limited by a lack of a practical biological reporter of abrogated DNA damage repair for drug development programs. H2AX phosphorylation as performed by either flow cytometry or 96-well immunocytochemistry allows the investigator to efficiently determine the effects of RT and drug therapy on DNA damage repair [28].

In this report, we describe a high-content approach to identifying novel inducers and/or inhibitors of DNA damage repair with radiosensitizing activity by performing high content screening of increased γ-H2AX after radiation. Analysis of the chemical structures of the two compounds that were positive on both primary and secondary screening assays showed significant structural similarity. Based on structural similarity to tripine, we hypothesized that these agents were ribonucleotide reductase inhibitors [29]. One prior report demonstrated that substituted 20acylpyridine-α-(N)-Hetarylhydrazones related to MS0019266 and MS0017509 inhibit ribonucleotide reductase in Ehrlich tumor cells and mouse leukemia L1210 cell growth in culture [29]. In this report, we demonstrated that both MS0019266 and MS0017509 have physiologically significant effects on DNA damage repair, cell cycle arrest, tumor cell proliferation and apoptosis. Although we hypothesize that the radiosensitizing effects of MS0019266 and MS0017509 is due to inhibition of ribonucleotide reductase resulting in impaired DNA double strand break repair, an indirect effect related to redistribution of cells within the cell cycle cannot be ruled out.

Since MS0019266 was slightly more effective at inducing apoptosis and cytotoxicity in prostate cancer cells than MS0017509, we further investigated its effect on gene expression. We demonstrated no effect of MS0019266 on ribonucleotide reductase gene expression. This is consistent with prior studies that demonstrate that compounds related to MS0019266, such as triapine, inhibit ribonucleotide reductase by generating reactive oxygen species [30]. However, in addition to reducing ribonucleotide reductase activity, MS0019266 significantly reduced expression of several genes related to cell cycle arrest and mitosis, including polo-like kinase 1, kinesin family member 20a, cyclin B1 and aurora kinase A. Treatment with either MS0019266 or MS0017509 dramatically reduced polo-like kinase-1 protein levels. It is not currently known whether MS0019266 or MS0017509 are direct inhibitors of polo-like kinase 1 or whether these compounds are non-specific inhibitors of mitotic proteins through effects on cell cycle. Polo-like kinase-1 plays a key role in cell cycle, including centrosome maturation and G2/M transition, and is a promising antitumor target [31]. Recent preclinical data suggests that polo-like kinase-1 inhibitors have radiosensitizing activity [32]. Search of the Ingenuity Pathways Analysis database demonstrates no known direct link between ribonucleotide reducase and polo-like kinase-1 (accessed July 17, 2010).

The *in-vitro* data were validated in two whole organism systems to assess *in vivo* antitumor efficacy and toxicity. The Drosophila model of MEN2 has been successfully used to study candidate compounds to treat MEN2B, including ret inhibitor ZD6474. Although there is no evidence that MS0019266 is a specific inhibitor of ret, we used the Drosophila model as a novel therapeutic discovery tool to demonstrate antitumor activity at doses without whole organism toxicity. Further mammalian data demonstrates no evidence of lethal systemic toxicity at doses that were active *in vitro* and in *Drosophila* (unpublished data). Taken together, MS0019266 and related compounds are promising anti-tumor and radiosensitizing compound that is a candidate for further development.

This study provides evidence that high-content screening as a feasible approach to developing agents that inhibit DNA damage repair. In this screen, we identified 2 compounds that are effective ribonucleotide reductase inhibitors, a well validated biological target for radiosensitization. Based on this preliminary experience, screening a larger chemical library may identify additional agents that specifically inhibit DNA damage repair. Other approaches to high-throughput screening for radiosensitizers have been recently described. Lally, et al performed a chemical library screen to identify agents that decreased cell viability of U251 glioma cells following radiation and drug therapy using a 96 well MTS assay [3]. They identified NS-123, which appears to inhibit DNA damage repair. Similarly, a 96-well colony forming assay was developed with the goal of identifying radiosensitizers [33]. In addition to providing information on DNA damage repair, this 96-well γ-H2AX assay could potentially be multiplexed for a high-content readout [6]. Our protocol utilized pooling of compounds, which is a commonly used approach to improve efficiency of resource intensive high-content screening protocols [34]. However, this strategy has the potential risks of additivity, synergy and antagonism that could result in both false positives and false negatives. We eliminated the risk of false positives through subsequent interrogation of individual compounds. A further disadvantage of pooled compound experiments is that the DMSO concentration is higher to achieve adequate drug concentrations and this is another potential confounding variable. To address the risk of false negatives associated with pooling, we propose subsequent screening of individual compounds from a larger chemical library that can be justified by our success in this pilot screen. In conclusion, MS0019266 and MS0017509 are promising compounds that may be candidates for further development as radiosensitizing compounds as inhibitors of ribonucleotide reductase and polo-like kinase 1.

## Methods

### Cell Lines and Cell Culture

We used one well characterized prostate cancer cell line (DU145) for screening and biological analysis and confirmed results in three human head and neck squamous carcinoma cancer (HNSCC) cell lines (SQ-20B, SCC61, SCC-25) and one prostate cancer cell line (PC-3) with known differences in radiosensitivity. DU145 and PC-3 cells were purchased from ATCC and was maintained in F-12K medium containing 10% fetal bovine serum and 1% penicillin/streptomycin. HNSCC cell lines were kindly provided by Dr. Aparna Ranganathan and maintained in Dulbecco’s modified Eagle’s medium/F-12 supplemented with 20% fetal bovine serum, 0.4 µg/ml hydrocortisone, 4 mM L-glutamine and 1% penicillin/streptomycin. Cells were maintained at 37°C and 5% CO_2_.

### Experimental Treatment

Cells were trypsinized from stock cultures, subcultured and grown to 50–70% confluence in 96-well tissue-culture treated glass bottom plates (Whatman Inc, Piscataway, NJ). Experimental radiation was performed using a Varian 600c clinical linear accelerator at a dose rate of 3.5 Gy/min and cells were immediately returned to the cell incubator. For each screening experiment, the chemical inhibitors were added to the media at the desired concentration for 4 hours. The final DMSO concentration were significantly less than 1% with single compound experiments had DMSO concentrations less than 0.1%. Ionizing radiation (4 Gy) and hydroxyurea (200 µM for 4 hours) were used as positive controls.

### Immunocytochemistry

We have previously described a 96-well immunocytochemistry assay for γ-H2AX [28]. Briefly, cells were treated with radiation and fixed in 4% paraformaldehyde for 20 minutes after IR and/or drug therapy permeabilized with 100% ice cold methanol for 3 hours. Subsequent PBS washes, blocking and staining steps were performed on the Tecan Freedom Evo liquid handling robot. Prior to primary antibody staining, cells were blocked using 3% normal goat serum. Cells were incubated with anti-phospho-H2AX polyclonal antibody (Cell Signaling, Danvers, MA) at 1∶1000 dilution for 1 hour at room temperature, washed and stained with Alexa 488 Fluor secondary antibody (Invitrogen, Carlsbad, CA) at 1∶600dilution for 60 minutes at room temperature [20]. Washes were performed with PBS and nuclear staining was performed using DAPI at 1 µg/mL. The wells are refilled with PBS and cells were imaged under fluorescent microscope.

### Semiautomated Microscopy and Analysis

The plates were imaged on an Olympus IX70 inverted confocal microscope with a LUCPLAN x40/0.6 numerical objective (Olympus, Center Valley, PA), Ludl focus motor, X-Y stage and Uniblitz shutters. Image acquisition was performed with a cooled Evolution QEi monochrome digital CCD camera (Media Cybernetics, Maryland, USA) operating at a maximum resolution of 1392×1040 pixels at 12 bits, with image transfer rate at 10 fps via FireWire (IEEE 1393) digital interface. The software used to visualize the images was InVitro Version 3.1.1 (Media Cybernetics, Maryland, USA) on an Intel Pentium dual core processor with a 30 inch wide screen monitor. After initial user intervention to determine the approximate plane of the cells throughout the plate, the automated software obtains images at two sites per well each 50 µm from the center of the well with 75 ms exposure time per image. Each well was focused over a ±150 µm range, and each site per well was focused over a ±20 µm range. Each field contains 7 different focal planes in the z-dimension, with a step size of 1 µm in between planes. Images were acquired under standardized brightness and contrast settings.

Subsequently, images for each cell were visually screened using ImageJ (National Institutes of Health, Bethesda, USA). Positives were cells with significantly increased γ-H2AX after treatment compared to DMSO only control wells.

### High-content Screening Design

The general algorithm for this preliminary screen is shown in Fig. S5. Briefly, DU-145 cells were treated with control (DMSO alone or hydroxyurea) or mixtures of 30 unknown compounds from a commercially available 14,400 compound chemical library (Chembridge, San Diego, CA) for 4 hours. This library was chosen for its structural diversity and availability of compounds for secondary assay and validation. Cells were stained for H2AX phosphorylation as described above. To determine the specific compound contributing to the phenotype observed in cells treated by compound mixtures, we performed further analysis of mixtures of 6 compounds followed by testing of individual compounds.

### Cell Viability Assay

As the initial secondary screen, inhibitory effects of compounds was evaluated by 3-(4, 5-dimethylthiazol-2-yl)-5-(3-carboxymethoxyphenyl)-2-(4-sulfophenyl)-2H-tetrazolium, inner salt (MTS; Promega, Madison, WI) assay. Briefly, cells were seeded in 96-well tissue culture plates at a density of 3×10^3^cells per100 µL/well. Four wells were assigned to each experimental treatment. After 24 hours for attachment, the compounds were added in a range of concentrations as single agent (0–10 µM). After 72 hours of treatment, 10 µL of MTS solution was added to each well for two hours incubation. Absorbance at 490 nm was recorded using microplate reader.

### Clonogenic Assay

Clonogenic survival curves were determined as follows. 1×10^3^cells were plated in 6-well cell culture plate. Compounds were added to cells 24 h later and irradiation was administered 24 h after drug treatment. Immediately after irradiation, the cultures were returned to the incubator and drug treatment was continued for a total of 72 hours. Subsequently, drug-containing media was removed and replaced with drug-free media. Since DU145 cells grow relatively slowly, cells were fixed and stained with Giemsa at 14 days after treatment. In contrast, SQ20B cells grow more rapidly and were ready for fixation and Giemsa staining at 10 days. Only colonies of >50 cells were scored as survivors. All data points are the results (mean ± SD) of 3–4 experiments. To compare cell survival between groups, a two-sample t test with unequal variances was utilized using Stata 8.0.

### Immunoblotting Analysis

Treated cells were washed with cold PBS and lysed in lysis buffer (Cell Signaling) using a standard protocol. The protein concentration was analyzed by a protein assay kit with bovine serum albumin standards according to the manufacturer’s instructions (Bio-Rad Laboratories, Hurcules, CA). Cell lysate was separated by SDS-PAGE and transferred onto a nitrocellulose membrane (Hybond-C, Amersham Pharmacia Biotech, Inc., Piscataway, NJ). Following blocking with PBS-Tween-20 containing 5% nonfat dry milk for 1 h, membranes were incubated overnight at 4°C with anti–phospho-H2AX antibody, anti-cleaved caspase 3 antibody, anti-RRM1 antibody, anti-RRM2 antibody, anti-BRCA1 antibody and anti-PLK1 antibody followed by incubation with horseradish peroxidase– conjugated secondary antibody. β-actin was used as loading control. All antibodies for *in vitro* experiments were purchased from Cell Signaling Technology except RRM1 and RRM2 (Santa Cruz, Santa Cruz, CA). Immunoreactive bands were detected by an enhanced chemiluminescence kit.

### Annexin V Assay for Apoptosis

1×10^5^cells were plated in P25 flasks. Compound was added to cells 24 hr later. At 72 h after treatment, cells were trypsinized and washed with cold PBS. Flow cytometry analysis was performed after cells were stained with both Annexin V and propidium iodine (PI) for 15 minutes at room temperature.

#### Cell proliferation and cell cycle assay

For bromodeoxyuridine (BrdU) experiments, cells were incubated with BrdU at a final concentration of 10 µM in cell culture medium after being treated with compound at desired time point. Then cells were fixed, permeabilized and stained with anti-BrdU antibody according to a commercially available BrdU flow cytometry staining protocol (BD Pharmingen, San Diego, CA). DNA content was stained by incubation of the cells with 7-AAD solution. Cell suspensions were analyzed with a BD FACSCalibur flow cytometry. Data were analyzed with CellQuest software.

### DNA Microarray

We performed GeneChip expression analysis to determine the function of lead compounds. Total RNA was isolated with the RNeasy Mini Kit (QIAGEN) from DU145 prostate cancer cells at 8 hours after drug treatment. RNA quality was assessed by agarose gel electrophoresis demonstrating a 28S band that is 2 times more intense than 18S ribosomal RNA. Synthesis of cDNA from total RNA and hybridization/scanning of microarrays were performed with Affymetrix GeneST1.0 array as described in the GeneChip manual. The raw intensity data were extracted from the GeneChip using Gene Expression Console (Affymetrix Inc.) and normalized across the chips by log scale robust multi-array analysis (RMA) method and the quality control of each chip data was performed by investigating overall intensity values for all probes, negative and positive controls [35]. To identify significantly differentially expressed genes between groups such as treated vs. control samples, Significance Analysis of Microarray (SAM, version2.1) was carried out [36]. SAM is a widely-used microarray differential analysis tool that correlates gene expression data to a wide variety of clinical parameters and uses permutation to estimate False Discovery Rate for multiple testing. The cutoff of FDR q value for SAM test was set to 0.1. The gene expression data was also integrated with Gene Ontology functional database using DAVID to perform functional enrichment for better interpretation of gene expression profiles in the context of biological function [37]. All data is MIAME compliant and the raw data was deposited in ArrayExpress (accession number E-MTAB-845).

### Ribonucleotide Reductase Assay

RR activity was measured utilizing the CDP assay method as previously described [30]. DU145 cells treated with MS0019266 (10 µM), MS0017509 (10 µM), hydroxyurea (200 µM) or vehicle control were washed with cold PBS and detached by trypsin and a cell scraper. Cells were transferred into a 15-mL tube and pelleted by centrifugation at 300 *g* for 10 min at 4°C. The pellets were washed again with PBS. One volume of low salt homogenization buffer (10 mM of HEPES, pH 7.2, with 2 mM of DTT) was added to the cell pellets. The cell suspension was homogenized by Pyrex tissue grinder (20 times up and down on ice). After homogenization, another 1x vol. of high salt buffer (1 M of HEPES, pH 7.2, with 2 mM of DTT) was added. The cell suspension was mixed by pipette (20 times up and down on ice). Cell debris were removed by centrifugation at 16,000 *g* at 4° for 20 min. The supernatant was passed through a Sephadex G25 spin column, pre-equilibrated with buffer (50 mM of HEPES, pH 7.2, 2 mM of DTT) to remove endogenous nucleotides. Protein concentration was measured on NanoDrop ND-1000 spectrophotometer (OD 280). The reaction mixture in a final volume of 50 µl contained the following: [^3^H]CDP (0.5 µCi;), HEPES (pH 7.2) (50 mM), DTT (6 mM), magnesium acetate (4 mM), ATP (2 mM), CDP (0.05 mM), and a specific amount of cell extract. The incubation time for the reaction was 20 min. [^3^H]dCDP was dephosphorylated by phosphodiesterase. [^3^H]C and [^3^H]dC were separated with a C_18_ ion exchange column by HPLC and detected by ß-RAM radioactive detector. The reaction was linear during the process.

### Multiple Endocrine Neoplasia Type 2 (MEN2) Whole Organism Drosophila Model

To efficiently assess the whole organism toxicity and antitumor activity of MS0019266, we used a well-established Drosophila model of MEN2 [38]. Compound MS0019266 dissolved in DMSO was mixed in melted fly food at ∼55°C and 1 ml allowed to set in a 5 ml plastic vial. 20–40 embryos of the indicated genotype (*ptc> dRet^MEN2B^* ) were transferred into each vial and allowed to develop at 25°C. DMSO at maximal concentrations of 0.2% in fly food was used as control. Number of pupae and adults were counted after ∼2 weeks to establish viability percentage.

*dRet^MEN2B^* mediated eye tumors were induced using the eyeless-FLP MARCM system. Using this strategy, FLP-mediated mitotic recombination initiated expression of oncogenic *dRet^MEN2B^* in small groups of cells mixed with wild type tissue. Clonal propagation of the *dRet^MEN2B^* expressing cells outcompeted adjacent wildtype cells leading to adult eyes showing overgrowth phenotype. For the eyeless-FLP MARCM experiments, *y,w,eyFLP1; Act5C>y^+^>Gal4, UAS-GFP; P[FRT]82B tub-Gal80* female virgins were crossed to *P[FRT]82B UASdRet^MEN2B^* males and their progeny raised in control or drug containing food. Enclosed adults were frozen and eyes imaged using standard scanning electron micrograph (SEM) procedures.

## Supporting Information

Figure S1 **MS0019266 treated SQ20B, SCC-61 and SCC-25 cells were assessed for cell viability using MTS assay.** After 72 hours of treatment with MS0019266 (10 µM) resulted in a significant decrease of cell viability in a dose-dependent compared to vehicle controls. Results are reported as means ±SEM and normalized to control (*P<0.05, **P<0.01; vs. control as determined by two-tailed Student’s *t*-test).
(TIF)

Figure S2 **MS0019266 treated PC-3 cells were assessed for cell viability using MTS assay.** After 72 hours of treatment with MS0019266 (10 µM) resulted in a significant decrease of cell viability in a dose-dependent compared to vehicle controls. Results are reported as means ±SEM and normalized to control (*P<0.05, **P<0.01; vs. control as determined by two-tailed Student’s *t*-test).
(TIF)

Figure S3 **Colony formation assay performed on SQ20B cells. MS0019266 (0.15 µM for 24 hours) significantly reduced clonogenic survival in SQ20B head and neck cancer cells subsequently treated with ionizing radiation.** Results are the average of experiment and each experiment was done in triplicate (n = 3).
(TIF)

Figure S4 **Structural similarity between MS0019266 and 3-aminopyridine-2-carboxaldehyde thiosemicarbazone (3-AP, triapine).**
(TIF)

Figure S5 **Chemical library screening algorithm.** Based on extensive preliminary data, persistence of γ-H2AX at 4 hours after treatment with drug therapy was identified as a robust phenotypic marker of unrepaired DNA damage and tumor cell sensitivity. The γ-H2AX immunocytochemistry assay has been developed and validated on single glass coverslips. Subsequently, an automated γ-H2AX immunocytochemistry assay was developed using 96-well glass bottom plates and robotic liquid handling. The initial chemical library screen was conducted with 30 compound mixtures from the 14,400 compound Chembridge library. Images from 96-well plates were captured using a CCD camera and analyzed off-line. The chemical compound resulting in significant persistence of γ-H2AX was identified using mixtures of 6 compounds and subsequently single compounds. Highly effective single compounds were assessed by MTT cell viability and clonogenic assays as secondary screens.
(TIF)
