# Correction: γ-H2AX Kinetics as a Novel Approach to High Content Screening for Small Molecule Radiosensitizers

**DOI:** 10.1371/annotation/0d7595f1-c719-4ed9-bef0-8308f4cac7e3

**Published:** 2012-09-28

**Authors:** Shibo Fu, Ying Yang, Tirtha K. Das, Yun Yen, Bing-sen Zhou, Ming-Ming Zhou, Michael Ohlmeyer, Eric C. Ko, Ross Cagan, Barry S. Rosenstein, Shu-hsia Chen, Johnny Kao

There was an error in the third author's name.

The correct name is: Tirtha K. Das.

The correct citation is: Fu S, Yang Y, Das TK, Yen Y, Zhou B-s, et al. (2012) γ-H2AX Kinetics as a Novel Approach to High Content Screening for Small Molecule Radiosensitizers. PLoS ONE 7(6): e38465. doi:10.1371/journal.pone.0038465 

The correct Author Contributions are: Conceived and designed the experiments: SF TKD Y. Yen RC JK. Performed the experiments: SF Y. Yang TKD BZ JK. Analyzed the data: SF TKD MO MMZ ECK BSR SHC JK. Contributed reagents/materials/analysis tools: SF Y. Yen MMZ MO RC BSR SHC JK. Wrote the paper: SF TKD BZ ECK JK. 

