# Supplementary figures and images for: γ-H2AX Kinetics as a Novel Approach to High Content Screening for Small Molecule Radiosensitizers
Source: PLoS One. 2012 Jun 29;7(6):e38465. doi: 10.1371/journal.pone.0038465 (PMC3387170; doi:10.1371/journal.pone.0038465)

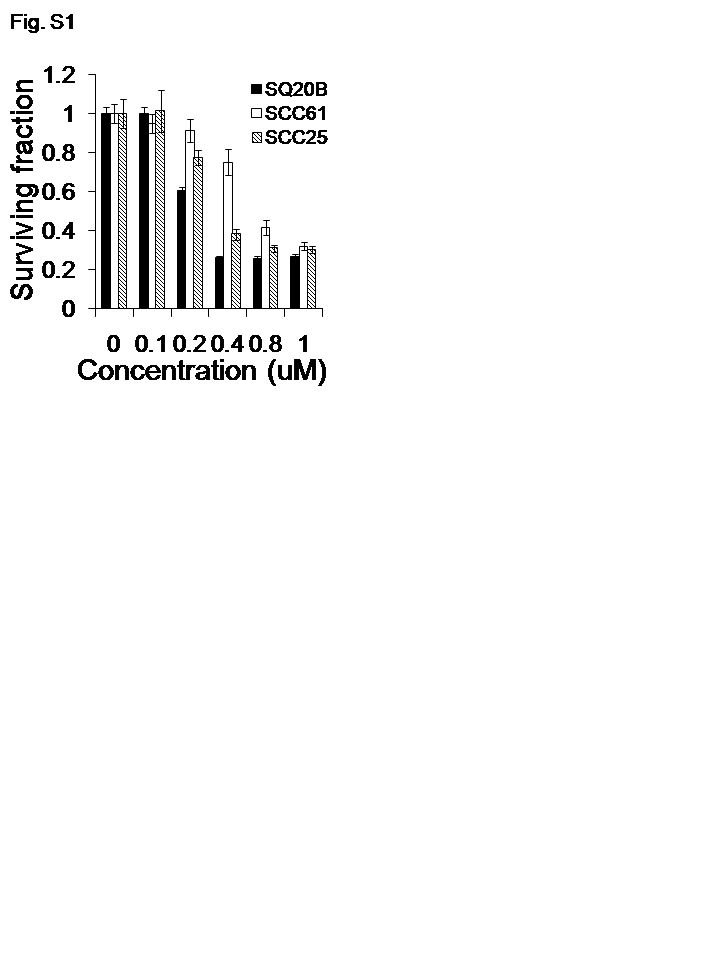

Supplement: Figure S1 — MS0019266 treated SQ20B, SCC-61 and SCC-25 cells were assessed for cell viability using MTS assay. After 72 hours of treatment with MS0019266 (10 µM) resulted in a significant decrease of cell viability in a dose-dependent compared to vehicle controls. Results are reported as means ±SEM and normalized to control (*P<0.05, **P<0.01; vs. control as determined by two-tailed Student’s t-test). (TIF) [file pone.0038465.s001.tif]

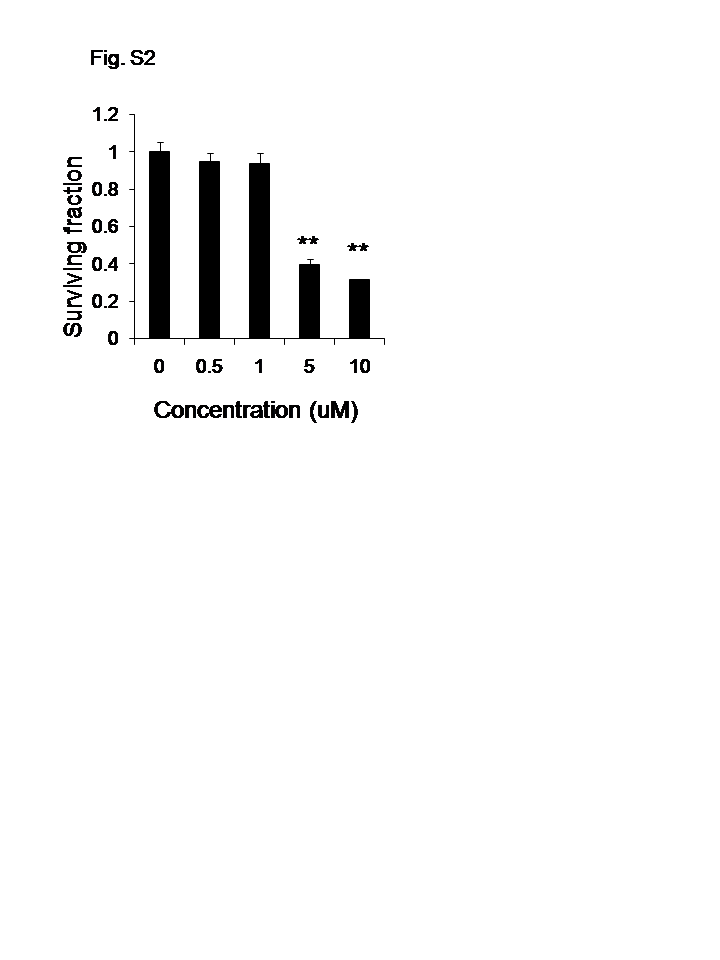

Supplement: Figure S2 — MS0019266 treated PC-3 cells were assessed for cell viability using MTS assay. After 72 hours of treatment with MS0019266 (10 µM) resulted in a significant decrease of cell viability in a dose-dependent compared to vehicle controls. Results are reported as means ±SEM and normalized to control (*P<0.05, **P<0.01; vs. control as determined by two-tailed Student’s t-test). (TIF) [file pone.0038465.s002.tif]

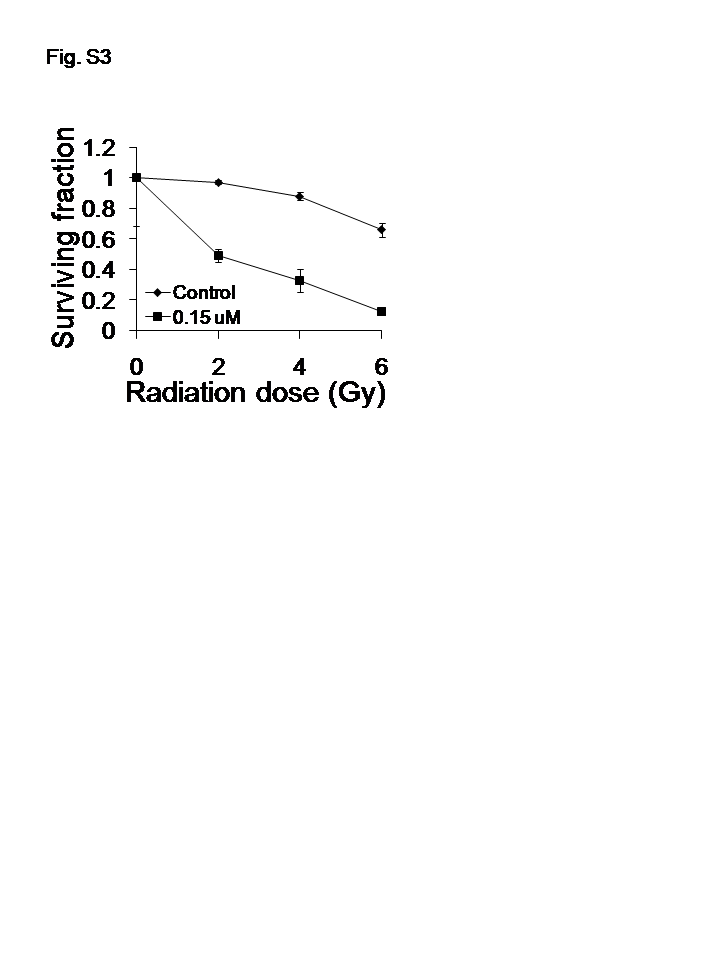

Supplement: Figure S3 — Colony formation assay performed on SQ20B cells. MS0019266 (0.15 µM for 24 hours) significantly reduced clonogenic survival in SQ20B head and neck cancer cells subsequently treated with ionizing radiation. Results are the average of experiment and each experiment was done in triplicate (n = 3). (TIF) [file pone.0038465.s003.tif]

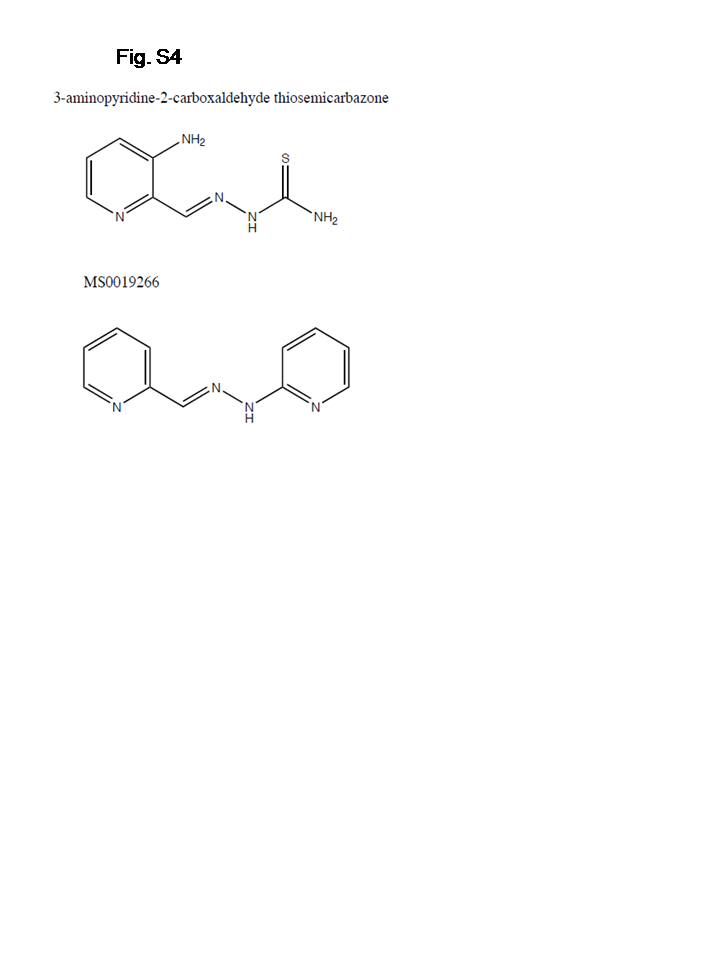

Supplement: Figure S4 — Structural similarity between MS0019266 and 3-aminopyridine-2-carboxaldehyde thiosemicarbazone (3-AP, triapine). (TIF) [file pone.0038465.s004.tif]

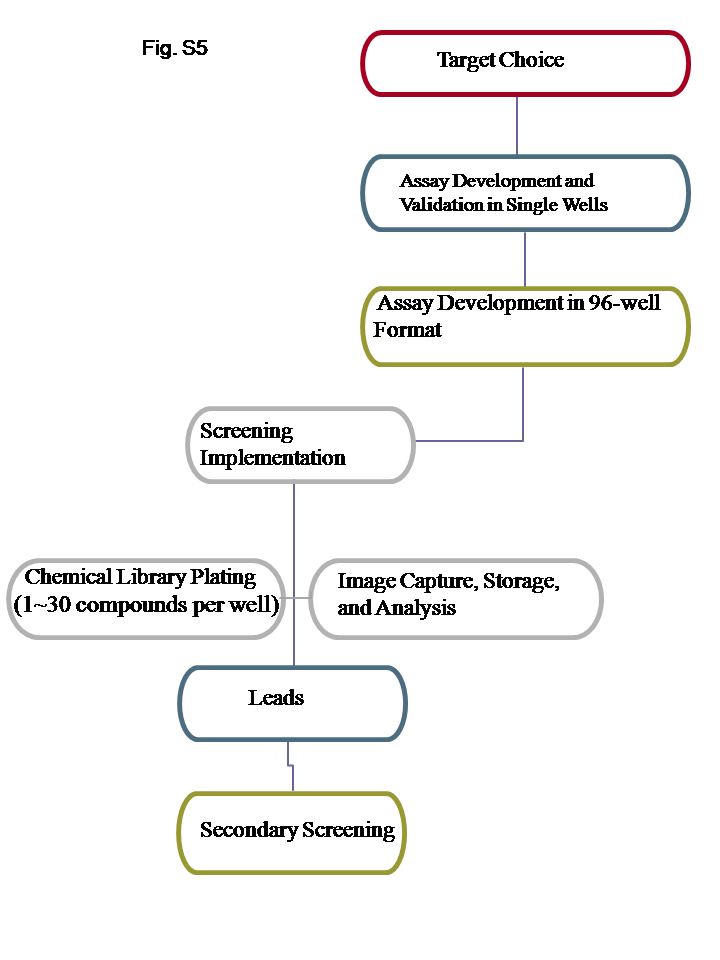

Supplement: Figure S5 — Chemical library screening algorithm. Based on extensive preliminary data, persistence of γ-H2AX at 4 hours after treatment with drug therapy was identified as a robust phenotypic marker of unrepaired DNA damage and tumor cell sensitivity. The γ-H2AX immunocytochemistry assay has been developed and validated on single glass coverslips. Subsequently, an automated γ-H2AX immunocytochemistry assay was developed using 96-well glass bottom plates and robotic liquid handling. The initial chemical library screen was conducted with 30 compound mixtures from the 14,400 compound Chembridge library. Images from 96-well plates were captured using a CCD camera and analyzed off-line. The chemical compound resulting in significant persistence of γ-H2AX was identified using mixtures of 6 compounds and subsequently single compounds. Highly effective single compounds were assessed by MTT cell viability and clonogenic assays as secondary screens. (TIF) [file pone.0038465.s005.tif]
